# Therapeutic potential of tucidinostat, a subtype-selective HDAC inhibitor, in cancer treatment

**DOI:** 10.3389/fphar.2022.932914

**Published:** 2022-09-02

**Authors:** Yichen Sun, Jing Han Hong, Zhiqiang Ning, Desi Pan, Xin Fu, Xianping Lu, Jing Tan

**Affiliations:** ^1^ State Key Laboratory of Oncology in South China, Collaborative Innovation Center of Cancer Medicine, Sun Yat-sen University Cancer Center, Guangzhou, China; ^2^ Department of Laboratory Medicine, Guangzhou First People’s Hospital, School of Medicine, South China University of Technology, Guangzhou, China; ^3^ Cancer and Stem Cell Biology Program, Duke-NUS Medical School, Singapore, Singapore; ^4^ Shenzhen Chipscreen Biosciences Co., Ltd., Shenzhen, China

**Keywords:** tucidinostat, HDAC, cancer, immunotherapy, lymphoma

## Abstract

Histone deacetylase (HDAC) is one of the most characterized epigenetic modifiers, modulating chromatin structure and gene expression, which plays an important role in cell cycle, differentiation and apoptosis. Dysregulation of HDAC promotes cancer progression, thus inhibitors targeting HDACs have evidently shown therapeutic efficacy in multiple cancers. Tucidinostat (formerly known as chidamide), a novel subtype-selective HDAC inhibitor, inhibits Class I HDAC1, HDAC2, HDAC3, as well as Class IIb HDAC10. Tucidinostat is approved in relapsed or refractory (R/R) peripheral T-cell lymphoma (PTCL), advanced breast cancer and R/R adult T-cell leukemia-lymphoma (ATLL). Compared with other HDAC inhibitors, tucidinostat shows notable antitumor activity, remarkable synergistic effect with immunotherapy, and manageable toxicity. Here, we comprehensively summarize recent advances in tucidinostat as both monotherapy and a regimen of combination therapy in both hematological and solid malignancies in clinic. Further studies will endeavor to identify more combination strategies with tucidinostat and to identify specific clinical biomarkers to predict the therapeutic effect.

## 1 Introduction

Epigenetics is the process that causes reversible changes in gene expression without genetic changes (Cavalli and Heard, 2019), which mainly includes histone modification, chromatin remodeling and DNA methylation. Enzymes that regulate epigenetics can be classified into three groups: writers that catalyze the addition of epigenetic marks to histone or DNA, such as histone acetyltransferases (HATs) and DNA methyltransferases (DNMT); readers that identify and interpret those modifications, such as bromodomain proteins; and erasers that catalyze the removal of epigenetic marks, such as HDACs and histone demethylases (Biswas and Rao, 2018).

Aberrant epigenetic modifications play important roles in tumorigenesis and are frequently found in human malignancies. Recurrent mutations in chromatin modifiers are most frequently occurring in many cancers, such as activating mutations in enhancer of zeste homolog 2 (EZH2) and inactivating mutations in CREB Binding Protein (CREBBP)/E1A binding protein P300 (EP300), lysine Methyltransferase 2D (KMT2D), SET domain containing 2 (SETD2), AT-rich interaction domain 1A (ARID1A), isocitrate dehydrogenase type 2(IDH2) (Cheng et al., 2019), suggesting that these epigenetic modifiers are ideal targets in therapeutic approaches. Chromatin modifiers known to be druggable include writers (such as EZH2, disruptor of telomeric silencing 1-like (DOT1L), and EP300), readers (such as bromodomain-containing proteins), and erasers (such as lysine demethylase 5 (KDM5) and HDACs). Several specific epigenetic-targeted agents for modifiers with gain-of-function (GOF) mutations have already been approved for clinical cancer treatment, such as EZH2 inhibitors (Cheng et al., 2019). Although targeting loss-of-function (LOF) mutations in CREBBP, KMT2D, and ARID1A is more challenging, many indirect targeted therapies and synthetic lethal agents are being evaluated. An example is potential drugs that exploit the synthetic lethal interaction between CREBBP and EP300 (Ogiwara et al., 2016), glutamate-cysteine ligase catalytic subunit (GCLC) and ARID1A (Ogiwara et al., 2019).

HDAC, an enzyme that removes the acetyl group from histones and nonhistone proteins, is one of the most studied epigenetic modifier (Mottamal et al., 2015). Acetylation of histone induces an open chromatin state to improve the binding of transcription factors to DNA, thus leading to increased gene expression (Bannister and Kouzarides, 2011). Acetylation of non-histone proteins including tumor suppressors and oncogenes (P53 and BCL6) modulates protein stability, protein interaction, or transcriptional activity (Pasqualucci et al., 2011). The acetylation of histone or non-histone protein is regulated by the balance between the activities of HDACs and HATs (Mondello et al., 2020). Based on cellular localization, function, and sequence homology, HDACs are classified into four classes: class I (HDAC1, HDAC2, HDAC3, and HDAC8); class II (HDAC4, HDAC5, HDAC6, HDAC7, HDAC9, and HDAC10), class III [NAD-dependent protein deacetylase sirtuin 1 (SIRT1)– SIRT7]; and class IV (HDAC11) (Dokmanovic et al., 2007). Imbalance between HDAC and HAT activities has been identified in hematological and solid malignancies, and are correlated with poor prognosis and survival (Yang et al., 2014). Therefore, regulation of HDAC and HAT are both important mechanisms during tumorigenesis and HDAC has become a promising target for cancer treatment.

HDAC inhibitors can be classified in four groups, including hydroxamates (vorinostat, belinostat, panobinostat), benzamide derivatives (entinostat, tucidinostat), cyclic peptides (romidepsin) and aliphatic acid. Currently, five HDAC inhibitors have been approved for clinical treatment of various hematological cancers and are being explored for their utility in solid tumors (Sermer et al., 2019). Vorinostat is the first HDAC inhibitor approved in 2006 for cutaneous T-cell lymphoma (CTCL), with an ORR of 29.7% (22/74) (Olsen et al., 2007). Romidepsin was approved for CTCL and PTCL in 2009 and 2011 respectively (Piekarz et al., 2011). Belinostat and Panobinostat were approved for CTCL and multiple myeloma (MM) respectively (Cheng et al., 2019). Tucidinostat was approved in R/R PTCL, advanced breast cancer and R/R ATLL. Tucidinostat was approved in China and Japan, and the other HDAC inhibitors mentioned above were approved by the FDA in the US. There are other HDAC inhibitors currently in clinical trials and have shown potential efficacy in cancer therapy, include entinostat, valproic acid.

**FIGURE 1:**
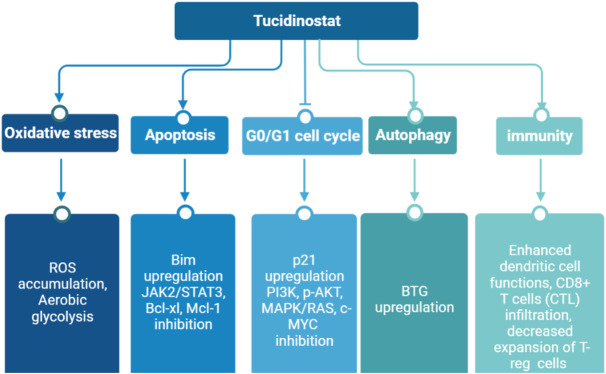
Epigenetic modifications caused by tucidinostat in multiple cancers. ROS: reactive oxygen species.

Tucidinostat is a novel benzamide HDAC inhibitor and inhibits Class I HDAC1, HDAC2, HDAC3, as well as Class IIb HDAC10 (Table 1) (Pan et al., 2014). Pre-clinical studies reveal that tucidinostat is efficacious against cancer cells *in vitro* and *in vivo*, supporting tucidinostat as a promising anti-cancer agent. Exposure to tucidinostat leads to a wide spectrum of biologic effects, including induction of apoptosis, oxidative stress, cell cycle arrest, autophagy and immune activation (Figure 1).

**TABLE 1:** Tucidinostat summary.

Drug name (generic)	Tucidinostat (Chidamide, Epidaza, Hiyasta)
Phase	II–III
	R/R peripheral T-cell lymphoma (CFDA, MHLW Japan)
Indication	Postmenopausal patients with hormone receptor-positive HER2-negative advanced breast cancer (NMPA)
	R/R adult T-cell leukemia-lymphoma (MHLW Japan)
Pharmacology	HDAC1, HDAC2, HDAC3 and HDAC10 inhibitor
Route of administration	Oral administration
Chemical structure	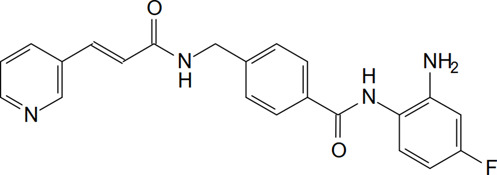

**FIGURE 2:**
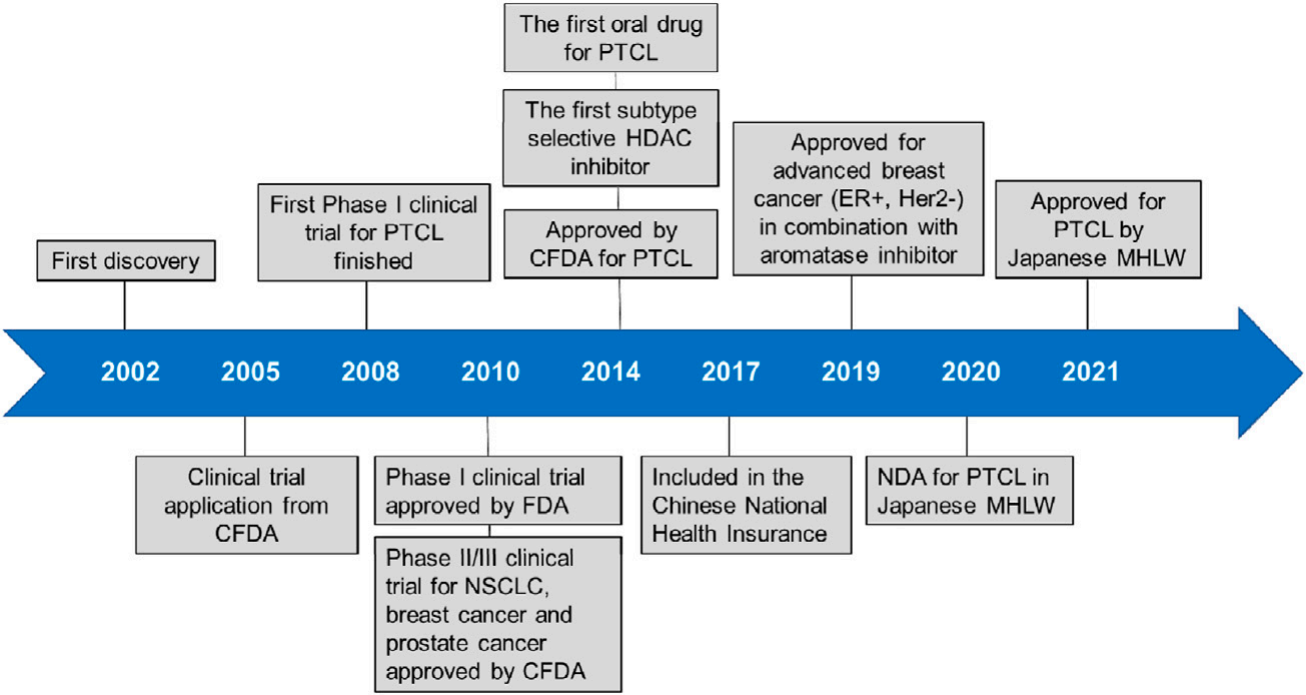
Timeline of tucidinostat development.

Tucidinostat is approved for R/R PTCL by China Food and Drug Administration (CFDA) and advanced breast cancer by National Medical Products Administration (NMPA) in China. Currently, it is approved for R/R ATLL and PTCL treatment by Ministry of Health, Labour and Welfare (MHLW) of Japan. Tucidinostat is the first HDAC inhibitor with subtype-selectivity and the first oral drug for PTCL worldwide. Moreover, it was the only HDAC inhibitor approved to date globally for treatment of solid tumor. Compared to other HDAC inhibitors, the superiority of tucidinostat for PTCL is reflected in many aspects such as effectiveness, synergistic effect with immunotherapy, safety, convenience and economy (Lu et al., 2016). More recently, it is being tested in multiple clinical trials as a single agent or in combination with cytotoxic chemotherapy and immunotherapy for cancer treatment. A timeline is shown in Figure 2, indicating the clinical development of tucidinostat in cancer therapy.

In this review, we focus on the clinical implications of tucidinostat in hematological malignancies and solid tumors, and provide a brief summary of preclinical and clinical trials of tucidinostat as a single treatment agent or in combination with other therapies.

## 2 Tucidinostat as a monotherapy

Since approved in 2014, tucidinostat was considered as a second-line and subsequent therapy for PTCL patients in China. Clinical trials and preclinical studies in multiple hematological malignancies and solid tumors is in progress.

### 2.1 Hematological malignancies

Tucidinostat has been studied as a monotherapy in several cancers and some other non-cancer diseases, including thrombocytopenia and acquired immune deficiency syndrome (AIDS). Tucidinostat is currently used for the treatment of T-cell lymphoma as a second-line therapy. While several clinical trials are ongoing, the development of tucidinostat is most advanced in peripheral T-cell lymphoma (PTCL). PTCL makes up 25%–30% of all Non-Hodgkin’s lymphoma (NHL) cases in China, much higher than that in Western countries of 10%–15% (Shi et al., 2015). In a phase II clinical trial with tucidinostat monotherapy, 28% (22/79) of R/R PTCL responded to treatment, 14% (11/79) achieved complete response (CR) and 14% (11/79) had partial response (PR) and the results led to the approval of tucidinostat in this indication by the CFDA. In 2017, a real-world multicenter efficacy and safety monitoring study to further evaluate the clinical practice value of tucidinostat in R/R PTCL patients was conducted in mainland China. For patients receiving tucidinostat monotherapy (*n* = 256), the overall response rate (ORR) and disease control rate (DCR) were 39.06% (100/256) and 64.45% (165/256), respectively(Shi et al., 2017). The updated data showed the median overall survival (OS) of patients receiving tucidinostat monotherapy was 433 days, suggesting a survival benefit (Liu et al., 2021). This large real-world and retrospective study further demonstrates a favorable efficacy and an acceptable safety profile of tucidinostat for R/R PTCL patients.

Due to heterogeneous features, nearly 50% of PTCL are unclassifiable and categorized as peripheral T-cell lymphomas, not otherwise specified (PTCL-NOS). Targeted sequencing of actionable biomarkers such as histone modifier genes are performed in PTCL-NOS to meet the urgent need to develop better therapeutic strategies. Ji et al. (2018) reported that PTCL-NOS patients with gene mutations in histone modifier genes, including EP300, KMT2D, and CREBBP, showed a remarkably increased response rate to tucidinostat monotherapy, as compared to those without mutations. In summary, while the clinical effect of tucidinostat for the treatment of PTCL has been elucidated, further mechanistic study and biomarker identification for patient stratification need to be illustrated in the future.

The efficacy of tucidinostat for B-cell lymphoma was recently reported. DLBCL is the most common subtype of NHL, comprising 30–40% of all new diagnoses (Swerdlow et al., 2016). Approximately 60–65% DLBCL patients can be cured with standard immunochemotherapy, the combination of rituximab, cyclophosphamide, doxorubicin, vincristine and prednisone (R-CHOP), while the remaining 35–40% patients will experience R/R DLBCL with a dismal prognosis and limited treatment options. Therefore, the exploration of new therapeutic approaches for R/R DLBCL patients is urgently needed. A phase II clinical trial assessed the tucidinostat monotherapy in 20 R/R DLBCL patients with at least two previous therapies. The ORR was 25% (5/20) with CR achieved in 15% (3/20) and PR in 10% (2/20). The median PFS for patients who responded to tucidinostat was 16.1 months, much longer than that of non-responders (1.6 months). The CREBBP-deficient DLBCL cells were more sensitive to tucidinostat compared to CREBBP-proficient DLBCL cells. In a phase II clinical trial of tucidinostat in DLBCL patients (Sun et al., 2021), DLBCL patients with CREBBP-deficiency exhibited a better response to tucidinostat treatment. Therefore, CREBBP was identified as a potential predictive biomarker for future patient stratification. Mechanistic studies revealed that tucidinostat sensitivity was mediated through transcriptional inhibition of cell cycle progression. Identification of specific predictive biomarker and molecular mechanism allowed for precision tucidinostat treatment for specific R/R DLBCL patients (Sun et al., 2021). It was reported that HDAC inhibitors could potently induce hepatitis B virus (HBV) lytic cycle, suggesting potential implications for the treatment of HBV-associated cancers (Yang Y. et al., 2021). Meanwhile, another Phase II clinical trial aimed to investigate the effect of tucidinostat as a maintenance therapy in 30 patients with HBV Positive DLBCL, which is still on-going and recruiting eligible patients (NCT04661943).

### 2.2 Solid tumors and other malignancies

Before tucidinostat was approved by the CFDA, tucidinostat was already studied as a single agent to treat solid tumors in some preclinical studies. For instance, the anticancer effect of tucidinostat for colon cancer was demonstrated through inhibition of phosphoinositide 3-kinase (PI3K) and mitogen-activated protein kinase (MAPK)/Ras pathways (Liu et al., 2010). Ten hepatocellular carcinoma cell lines were shown to be sensitive to tucidinostat through upregulation of p21 expression. Bin Zhao reported that tucidinostat inhibited pancreatic cancer both *in vitro* and *in vivo* through regulating mitochondrial apoptosis (Zhao and He, 2015) and aerobic glycolysis (He et al., 2016).

Adenoid cystic carcinoma (ACC) is a malignant epithelial neoplasm with no single targeted drug. and the development of ACC was characterized by chromatin remodeling. The proliferation of ACC cells and cell-derived xenografts were significantly inhibited through cell cycle arrest in G2/M phase by increased acetylation of histone H3 and phosphorylation of Protein kinase B (AKT) protein due to use of tucidinostat (Yang et al., 2018). In the phase I study of tucidinostat with advanced solid tumors and lymphomas, one patient with adenoid cystic carcinoma of the submandibular gland achieved a partial response (Dong et al., 2012). There are ongoing clinical trials studying the efficacy of tucidinostat monotherapy in advanced head and neck adenoid cystic carcinoma (Table 2).

**TABLE 2:** Ongoing clinical trials of tucidinostat as monotherapy.

Num	Study Title	Dose of tucidinostat	Phase	Estimated enrollment	NCT number (reference)	Study Start Date
1	A Clinical Trial of the Low Dose Tucidinostat in the Management of Refractory ITP	2.5 mg, TIW; 5 mg, TIW	Phase II	30	NCT03838354 (Zhao et al., 2019)	1-Jun-19
2	Differences Between Tucidinostat Taken Daily and Twice a Week in Therapeutic Effect, Pharmacokinetics, Pharmacodynamics and EB Virus Activation	10 mg, QD; 30 mg, BIW	Phase II	24	NCT02878278	Sep-16
3	Tucidinostat for Advanced Cephalic and Cervical Adenocystic Carcinoma: Evaluation of Efficiency and Safety	30 mg, BIW	Phase II	30	NCT02883374	Nov-16
4	Maintenance Therapy of Tucidinostat in Patients With HBV Positive Diffuse Large B-cell Lymphoma	20 mg, BIW	Phase II	20	NCT04661943	30-Nov-20
5	Tucidinostat for Patients With Recurrent and Metastatic Epstein-Barr Virus (EBV)-Associated Solid Tumors	30 mg, BIW	Phase II	66	NCT03494634	11-Apr-18
6	Tucidinostat for Patients With Relapse or Refractory Diffuse Large B-Cell Lymphoma and Follicular Lymphoma	30 mg, BIW	Phase II	40	NCT03410004	15-Apr-18
7	Study of Tucidinostat as a Single-agent Treatment for Patients With Relapse or Refractory B-NHL	30 mg, BIW	Phase II	100	NCT03245905 (Sun et al., 2021)	25-Jan-18
8	Study of Tucidinostat for Steroid-resistant/Steroid-dependent Severe cGVHD	15 mg, BIW	Phase I, Phase II	20	NCT05140616	20-Aug-17

TIW, three times per week; BIW, twice per week; QD, every day; cGVHD, Chronic Graft-versus-Host Disease 31-May-21.

Tucidinostat is also investigated in some non-cancer diseases. In disorders with immune deregulation involved in the pathogenesis, low-dose HDAC inhibitors were reported to enhance the number and function of Foxp31 regulatory T (Treg) cells and to enhance immunosuppression and tucidinostat showed potential as a novel treatment for idiopathic thrombocytopenic purpura (ITP) in the clinic (Zhao et al., 2019). The clinical trial to investigate the safety and efficacy of low-dose tucidinostat to treat chronic graft-versus-host disease (cGVHD) is now ongoing and recruiting patients (Table 2).

## 3 Tucidinostat as a combination regimen

Currently, tucidinostat is used in combination therapy in most clinical trials, including in combination with chemotherapy, targeted therapy, radiotherapy, and immunotherapy, in both hematological malignancies and in solid tumors.

### 3.1 Tucidinostat in combination with chemotherapy

#### 3.1.1 Hematological malignancies

The multicenter real-world study in China showed that the ORR for R/R PTCL patients receiving tucidinostat combined with chemotherapy was 51.18% (87, 127), higher than that of monotherapy tucidinostat (39.06%). The results of a subgroup analysis showed that the ORRs for patients receiving tucidinostat combined with CHOP (cyclophosphamide, doxorubicin, vincristine, and prednisone)-like regimens, platinum-containing regimens, and other regimens were 53.13, 45.83, and 55.32%, respectively. This study illustrates that tucidinostat combined with chemotherapy may be a new treatment option for PTCL, especially for PTCL patients with an International Prognostic Index (IPI) ≥2 (Shi et al., 2017). Etoposide with CHOP (CHOEP) regimen is another commonly used treatment for PTCL patients. The updated data showed the median OS of patients receiving tucidinostat combination therapy was 463 days suggesting a survival benefit (Liu et al., 2021). Thus, a prospective, multicenter Phase Ib/2 study was conducted to evaluate the efficacy and safety of tucidinostat and CHOEP (Chi-CHOEP) in previously untreated patients with PTCL. The Chi-CHOEP regimen was well tolerated, and the observed adverse events were manageable. The results showed that the overall response rate was 60.2% (68/113), with a complete response rate of 40.7% (46/113) (Zhang et al., 2021). In order to compare the efficacy of chemotherapy plus tucidinostat vs. chemotherapy for patients with newly diagnosed PTCL, a study was carried out and data showed patients in chemotherapy plus tucidinostat group had superior progression-free survival (PFS) (*p* = 0.047). This study indicated the combination of chemotherapy and tucidinostat may provide a promising prospect for patients with newly diagnosed PTCL (Wang J. et al., 2022).

Compared to intensive chemotherapy, oral metronomic chemotherapy has been used as a less intensive and cost-effective treatment. A phase II clinical trial investigating the efficacy and safety of an all-oral regimen containing tucidinostat, prednisone, etoposide, and thalidomide (CPET) in untreated patients with angioimmunoblastic T-cell lymphoma (AITL) demonstrated an effective, tolerable, and economical choice for untreated AITL in a Chinese population (Wang et al., 2022c). The CR rate of CPET in AITL was 54.9% (28, 51), which was significantly higher than that of the standard regimen CHOP, 33.0% (Abouyabis et al., 2011). However, the efficacy population of CPET study in AITL was limited and further studies with a larger sample size might be required. Furthermore, several ongoing clinical trials were conducted to study tucidinostat-based oral metronomic chemotherapy (NCT03321890, NCT02879526).

More than 50% of DLBCL cases are elderly patients over 60 years old, which present with poor survival and intolerance to intensive chemotherapy. A phase II study was conducted to investigate the efficacy and safety of tucidinostat plus R-CHOP (CR-CHOP) in newly diagnosed DLBCL. Among 49 patients, the CR was 86%, with ORR achieving 94% (Zhang et al., 2020). The CR was higher than those of previous reports in Western countries (71–76%) (Pfreundschuh et al., 2008) and in China (72%) (Xu et al., 2019). Another encouraging finding of the study was the clinical efficacy of CR-CHOP in the Double-expresser lymphoma (DEL) phenotype of DLBCL. Another phase III, randomized, double-blind, placebo-controlled, multicenter study is currently ongoing in China (NCT04231448) to confirm the therapeutic efficacy of CR-CHOP in DEL DLBCL. To identify potential molecular biomarkers predictive of responses to CR-CHOP, whole exome sequencing (WES) and targeted sequencing were conducted in 36 patients. The results showed that mutations in KMT2D, but not in CREBBP/EP300, were associated with inferior clinical outcomes including inferior PFS and OS. Therefore, CR-CHOP is well tolerated and showed promising clinical activity in DLBCL (Zhang et al., 2020). For R/R DLBCL patients who are ineligible for intensive chemotherapies, tucidinostat with PEL Regimen (Prednisone, Etoposide, Lenalidomide) showed favorable efficiency and moderate safety profile (Wang et al., 2022b).

A phase II clinical trial assessed the combination of tucidinostat, cladribine, gemcitabine, and busulfan (ChiCGB) in 105 patients (60 with B-cell non-Hodgkin lymphomas (B-NHL), and 45 with T-cell, or natural killer/T-cell lymphoma (NKT) followed by autologous stem cell transplant (ASCT) (Ji et al., 2021). The 4-year OS was 86.1%, which is significantly higher than the OS of traditional therapies (less than 70%), as reported in previous studies (Stiff et al., 2013). The results showed that the ChiCGB is an efficient conditioning regimen to prevent relapse after transplant and lead to better long-term overall survival for lymphoma patients.

There are also several pre-clinical studies investigating the synergistic effect and molecular mechanism of tucidinostat in combination with chemotherapy. rituximab/chemotherapy resistant B-cell lymphoma (RRCL) patients are characterized with poor prognosis, while tucidinostat reverses the chemotherapeutic resistance through B-cell translocation gene 1 (BTG1)-mediated autophagy. Tucidinostat shows synergistic effect with chemotherapeutics, such as etoposide, cisplatin, gemcitabine, in RRCL cell lines (Xue et al., 2021). Zhang et al. showed that tucidinostat and doxorubicin exhibited a synergistic effect in 2 PTCL cell lines through increased DNA damage and apoptosis (Zhang et al., 2017). The low dose of tucidinostat was also reported to enhance the therapeutic effect of DNA-damaging agents (daunorubicin, idarubicin, and cytarabine) for recurrent/resistant acute myeloid leukemia (AML) as an alternative salvage regimen, especially those possessing stem and progenitor cells (Li et al., 2017).

In addition to PTCL and DLBCL, tucidinostat was investigated in several other hematological malignancies, such as MM, Myelodysplastic syndromes (MDS), and T-cell lymphoblastic lymphoma (T-LBL). MM is a plasma malignancy characterized by the accumulation of monoclonal plasma cells in the bone marrow (BM), high levels of monoclonal immunoglobulins in the serum and osteolytic bone lesions. Tucidinostat induced cytotoxicity in myeloma cells through apoptosis and cell cycle arrest. Importantly, tucidinostat suppressed osteoclast differentiation and resorption *in vitro* and tumor-induced bone loss *in vivo*. This study indicates the potential use of tucidinostat in the treatment of MM in the future (Huang et al., 2019). Sun et al. revealed that tucidinostat is effective in MM treatment through regulating levels of H3K27ac to increase the transcription of succinate dehydrogenase subunit A (*SDHA*), which is proven to be a potential prognostic factor of MM patients (Sun et al., 2020). MDS is a heterogeneous group of disorders characterized with peripheral blood cytopenia and dysplastic bone marrow, with a high risk of transformation to AML. Zhao et al. (2016) demonstrated that tucidinostat potently inhibited the tumorigenesis of MDS and AML cells through inhibiting Janus kinase 2 (JAK2)/signal transducer and activator of transcription 3 (STAT3) signaling. Tucidinostat was also shown to inhibit growth of leukemia cells (including promyelocytic leukemia, chronic myelogenous leukemia, and T lymphocytic leukemia cells) both *in vitro* and *in vivo* through intracellular reactive oxidative stress (ROS) accumulation (Gong et al., 2012). HDAC inhibitors showed significant therapeutic potential in T-ALL by epigenetic regulation of the transcription of neurogenic locus notch homolog protein 1 (NOTCH1) target genes, thus tucidinostat was studied in five patients with NOTCH1 and RAS/PTEN mutations among 43 T-LBL patients, using circulating tumor DNA (ctDNA) profiling test for diagnosis. The results showed that none of the five patients relapsed, while more than half of the patients (22/43, 51.2%) had R/R disease and none of R/R patients survived (Chen F. et al., 2020). However, further studies with more patients enrolled are required to clarify the efficacy of tucidinostat in T-LBL.

#### 3.1.2 Solid tumors

Many pre-clinical trials have investigated the efficacy of tucidinostat with chemotherapy in solid tumors. Gemcitabine is the first-line chemotherapy for pancreatic cancer, which is a highly lethal disease. It is reported that tucidinostat synergistically enhanced gemcitabine-induced cell death in pancreatic cancer cell lines through extensive DNA damage (Qiao et al., 2013). Cisplatin is another chemotherapeutic drug commonly used in several kinds of solid tumors. The combination of tucidinostat and cisplatin has been investigated in non-small cell lung cancer (NSCLC) cells (Zhou et al., 2014), adenoid cystic carcinoma cells (Yang et al., 2018) both *in vitro* and *in vivo*.

There are no reports of completed full clinical trials of tucidinostat in the combination with chemotherapy in solid tumors yet. Hu et al. reported a phase I trial of tucidinostat plus with paclitaxel and carboplatin in advanced non-small cell lung cancer in 2017 (Hu et al., 2016). The results showed that the combination regimen was well tolerated, and the related phase II trial (NCT01836679) is ongoing. Another phase II study reported the therapeutic efficacy of tucidinostat with cisplatin for advanced triple-negative breast cancer (TNBC). Single-agent platinum chemotherapy has been shown to induce an ORR of 31% (Waks and Winer, 2019), while the result in this trial showed an ORR of 26.7% (4/15), which indicates that the addition of tucidinostat did not improve the efficacy of cisplatin in the first-line treatment against advanced TNBC (Meng et al., 2021).

Multi-drug resistance (MDR) occurs usually during chemotherapy of solid tumors, which is the main reason for the treatment failure and cancer recurrence. Since HDAC plays an important role in multidrug resistance, HDAC inhibitors are potential regents to overcome chemotherapeutic resistance. Cao et al. (2021) reported the synergistic effect of tucidinostat and doxorubicin in MDR breast cancer cells, suggesting the potential role of tucidinostat to overcome chemoresistance in breast cancer.

### 3.2 Tucidinostat in combination with targeted therapy

Tucidinostat has been used with several kinds of drugs targeting small molecules in preclinical and clinical studies, such as inhibitors targeting DNMT, EZH2, aurora kinase A (AURKA), proteasome, and B-cell lymphoma 2 (BCL-2).

#### 3.2.1 Hematological malignancies

Decitabine, a DNA methylation inhibitor, is used to treat MDS and AML. It is reported that tucidinostat and decitabine showed a synergistic effect to inhibit cell growth of Hodgkin lymphoma through upregulating the expression of tumor suppressor genes *PU.1* and *Kruppel-like factor 4* (*KLF4*) (Jiang et al., 2017). Resistance to multiple agent chemotherapy is a common clinical challenge encountered in AML treatment. In recent decades, epigenetic treatment containing HDAC and DNMT inhibition has been a major breakthrough in AML. A single-arm, Phase I/2 study investigated the combination of tucidinostat, decitabine, cytarabine, aclarubicin, and granulocyte colony-stimulating factor (CDCAG) in patients with R/R AML. The ORR was 46.2% (43/93) with a 25.8% CR rate (24/93) and 20.4% patients achieved CR with incomplete blood count recovery (CRi). The CDCAG regimen showed good antileukemic activity and acceptable toxicity (Wang et al., 2020). Based on this study, the regimen was recommended by 2021 Guidelines of Chinese Society of Clinical Oncology (CSCO) hematological malignancies (ISBN: 9787117314411). In another phase II trial for R/R AML, tucidinostat, decitabine, cytarabine, idarubicin, and granulocyte-colony stimulating factor (CDIAG) were used in a double epigenetic priming regimen. The ORR was 42.9% (15/32) with the median OS time of 11.7 months, indicating a good antitumor effect of CDIAG regimen for AML patients (Yin et al., 2021).

EZH2, a histone methyltransferase, is one of the frequently mutated epigenetic genes in DLBCL. The EZH2 specific inhibitor SHR2554 is currently undergoing clinical trials for the treatment of R/R lymphoid neoplasms. The synergistic anti-proliferative efficacy between tucidinostat and SHR2554 was shown, through the downregulation of DNA replication initiator protein ORC1 (Wang X. et al., 2021).

In the study of tucidinostat as monotherapy in DLBCL, it was found that AURKA inhibitors could overcome tucidinostat resistance in tucidinostat resistant cells. Firstly, the molecular mechanism of tucidinostat in DLBCL was shown to act through the cell cycle machinery. Then AURKA inhibitors (Alisertib and VX680), identified through high-throughput drug screening, had shown synergistic effect with tucidinostat both *in vivo* and *in vitro*. Therefore, the combination of an AURKA inhibitor and tucidinostat is a novel therapeutic strategy for the treatment of R/R DLBCL (Sun et al., 2021).

Bortezomib (BTZ) acts as a proteasome inhibitor to inhibit protein degradation, and has been used for MM and mantle cell lymphoma. It was reported that tucidinostat corporately potentiates the antimyeloma effect of bortezomib partly through repressing autophagic degradation of ubiquitinated proteins (Xu et al., 2020). Like other targeted therapies, drug resistance also occurs commonly in MM patients. He et al. reported that tucidinostat could reverse bortezomib resistance through enhanced ROS production and DNA damage (He et al., 2021).

ABT199 (Venetoclax) is a selective BCL-2 inhibitor approved as a component of combination therapy for AML. It was reported that low-dose tucidinostat enhanced the anti-AML activity of ABT199, resulting in a better synergistic effect than with romidepsin. Tucidinostat enhanced the efficacy of ABT199 through increasing DNA double-strand breaks and unbalancing of anti- and pro-apoptotic proteins (Bim upregulation and Bcl-xl downregulation) (Chen K. et al., 2020).

#### 3.2.2 Solid tumors

The most well-known combination of tucidinostat with targeted therapy for solid tumors is the combination with exemestane for breast cancer. Jiang et al. conducted the first Phase III trial of tucidinostat and exemestane for postmenopausal patients with advanced, hormone receptor (HR)-positive breast cancer (ACE). 365 patients were recruited, 244 were assigned to tucidinostat group and 121 to the placebo group. The median PFS was 7.4 months in the tucidinostat group [ORR, 18% (45/244)] and 3.8 months in the exemestane monotherapy group [ORR, 9% (11/121)]. The results showed that the combination with tucidinostat improved the PFS of the exemestane monotherapy group with accepted adverse effect. Since drug resistance to endocrine therapy is a major challenge in the treatment of HR-positive breast cancer, the combination of tucidinostat and exemestane could be a new treatment option (Jiang et al., 2019).

There are also preclinical studies of tucidinostat in combination with targeted therapies for NSCLC to reverse resistance to drugs that include epidermal growth factor receptor tyrosine kinase inhibitor (EGFR-TKI) icotinib (Zhang N. et al., 2019) and the receptor tyrosine kinase anaplastic lymphoma kinase (ALK) inhibitor crizotinib (Ding et al., 2020).

### 3.3 Tucidinostat in combination with radiotherapy

Radiotherapy in combination with chemotherapy is recognized as the standard treatment for high-risk NKTCL, but systemic failure occurs in nearly 30% patients. Therefore, tucidinostat, as a novel HDAC inhibitor, was combined with radiotherapy and the clinical efficacy investigated. The phase II clinical trial of intensity-modulated radiation therapy (IMRT) followed by gemcitabine with or without tucidinostat in patients with high-risk early-stage NKTCL is ongoing (NCT04511351). Another preclinical study reported the synergistic effect of tucidinostat and radiotherapy in inducing cell apoptosis and suppressing cancer stemness through regulating mir375- Eukaryotic translation initiation factor 4 gamma 3 (EIF4G3) axis in lung squamous cell carcinomas (Huang et al., 2021). The radiotherapy sensitization effect of tucidinostat needs to be further investigated in more clinical and preclinical studies.

### 3.4 Tucidinostat in combination with immunotherapy

The conventional anticancer treatment strategies have been surgery, chemotherapy, and radiotherapy for decades. Currently, immunotherapy is taking a leading role in the field of cancer research. Cancer immunotherapy has been shown to improve the overall survival of patients with different types of cancers, especially for hematological malignancies. However, only a subset of patients could achieve response by anti-programmed cell death protein 1(PD-1) immunotherapy. It was recently uncovered that HDAC2-dependent regulation of programmed death-ligand 1 (PD-L1) nuclear localization leads to pro-inflammatory pathways and immune checkpoint gene control. This might have important suggestions for the combination of anti-PD-1 immunotherapy and HDAC inhibitors (Gao et al., 2020b). Tucidinostat was shown to induce immunogenicity including 1) enhanced immune-cell mediated cytotoxicity *in vitro* (Ning et al., 2012), 2) enhanced dendritic cell functions (Wei et al., 2021a), 3) enhanced CD8^+^ T cells (CTL) infiltration (McCaw et al., 2017), 4) decreased expansion of T-regulatory and myeloid-derived suppressor cells (Dai et al., 2021). Therefore, tucidinostat, as a novel HDAC inhibitor, has been applied in combination with immunotherapy to investigate the immune sensitization effect.

#### 3.4.1 Hematological malignancies

Rituximab is a monoclonal antibody against B-lymphocyte antigen CD20, which is expressed on the surface of B cells. Rituximab is commonly used to treat certain types of cancer, such as NHL and chronic lymphocytic leukemia (CLL). R-CHOP immunotherapy is the standard therapy for DLBCL, which has improved overall survival by 10–15%, compared to treated with CHOP alone (Lenz et al., 2008). Tucidinostat has been applied in clinical trials to improve the therapeutic efficacy of R-CHOP therapy, but the potency and the molecular mechanism of the combination of tucidinostat and rituximab remain unclear. Guan et al. investigated the synergism between tucidinostat and rituximab both *in vivo* and *in vitro*. They found that tucidinostat significantly overcomes rituximab-mediated down-regulation of CD20 and facilitates the rituximab-induced anti-cancer effect. CD20 expression is a potential biomarker to evaluate the clinical response in DLBCL patients to the combination treatment (Guan et al., 2020).

Interaction between PD-L1 and PD-1 have been identified as critical for the immune regulation of cancer progression. The specific inhibitors could activate the immune system to attack and eliminate cancer cells indirectly, which has achieved superior clinical responses with fewer side effects in a broad range of cancers (Brahmer et al., 2012; Herbst et al., 2016; Kato et al., 2019). T-cell NHL patients, who have Regulatory T lymphocytes (Treg) with high PD-1 expression are characterized with medium/high risk. After treatment with tucidinostat combined with chemotherapy, the PD-1 expression showed a significant loss, which did not occur after chemotherapy treatment alone. The phenomenon indicates that PD-1 expression is regulated by tucidinostat, although the mechanism is not clarified (Zuo et al., 2018). Zhang et al. identified that circulating PD-1 (+) cells are characterized with a decreased level of IFN-γ secretion and impaired cytotoxic activity, compared with PD-1 (−) cells of PTCL patients. The deficiencies could be recovered by tucidinostat through upregulation of adaptive immune-associated genes in PD-1 (+) cells (Zhang W. et al., 2019).

Wei et al. studied the effect of tucidinostat on circulating PD1 (+) cells from PTCL patients. After performing gene expression profile analysis of peripheral blood PD1 (+) cells, they found that the expression of genes associated with chemokine activity and chemotaxis function were enhanced in the CR patients. These findings suggested that tucidinostat may remodel the tumor microenvironment to an anti-cancer phenotype and have the potential to synergize with immune checkpoint inhibitors (Wei et al., 2021b).

A phase Ib/II trial was conducted to investigate the effect of tucidinostat in combination with anti-PD-1 antibody sintilimab in R/R extranodal natural killer/T cell lymphoma (ENKTL). The study consisted of a phase Ib for dose escalation and a phase II for expansion. In phase Ib, no dose limiting toxicity (DLT) were observed. In phase II, the results showed that the ORR was 58.3% (21/36) with CR of 44.4% (16/36) and PR of 13.9% (5/36). This study reported for the first time that tucidinostat with sintilimab showed a manageable safety profile and exhibited effective antitumor activity, which prompted the combination of epigenetic strategies and anti-PD1 antibody as a promising treatment option for R/R ENKTL (Gao et al., 2020a). One exploratory study investigating the efficacy and safety of sintilimab plus tucidinostat for patients with newly diagnosed ENKTL was untertaken and the combination yielded effective antitumor activity and manageable toxicities. It indicates the regimen might be a promising chemo-free induction therapeutic portion for newly diagnosed ENKTL, especially for early-stage patients (Yan et al., 2021).

The anti-PD1 antibody camrelizumab has been applied with another epigenetic inhibitor decitabine in R/R HL and up to 71% patients received CR (Nie et al., 2019). Wang et al. conducted the phase II study to assess the safety and efficacy of the regimen in combination with tucidinostat in decitabine-plus-camrelizumab resistant cHL patients. The results showed a high ORR (93%, 13/14) with CR rate of 43% (6, 14) and PR rate of 50% (7, 14). Therefore, the trial indicates that the addition of tucidinostat to decitabine-plus-camrelizumab was within an acceptable safety profile without triggering immune-related adverse events (Wang C. et al., 2021).

Histone deacetylases inhibitors (HDACis) played critical roles during the activation and differentiation of T helper (Th) cells (Boucheron et al., 2014; Ellmeier and Seiser, 2018). HDAC1 and HDAC2 inhibition induced the activation of CD4^+^ T cells and CD8^+^ T cell effector programme that was further enhanced by Th0 and Th1 cells (Boucheron et al., 2014). Th17 cells and Th17 cell-driven autoimmunity were also regulated by HDACs (Limagne et al., 2017). The clinical study investigated the new epigenetic regimen (Tucidinostat, Decitabine, and Thymalfasin) on AML patients and the ORR was 79.2% (19/24). During the treatment process, Th1 cells and CD3^+^CD4^−^CD8^+^ T cells increased, and Th17 cells decreased, which reflects the effective treatment-related immune effect. Consequently, the elevated ratio of Th1/Th17 could be a potential biomarker for prognosis evaluation (Xi et al., 2021).

CD38 is a transmembrane glycoprotein and functions as a receptor and adhesion molecule, which is highly expressed on MM cells. Therefore, anti-CD38 antibodies, such as daratumab, has been considered an effective therapy for MM patients (Van De Donk et al., 2018). It was reported that pan-HDAC and HDAC6 inhibitors could upregulate the expression of CD38 to increase the efficacy of anti-CD38 therapy (García-Guerrero et al., 2017; Hirano et al., 2018; García-Guerrero et al., 2021). The combinatory efficacy of the novel HDAC inhibitor tucidinostat with CD38 antibodies needs to be further evaluated in pre-clinical studies and clinical trials.

Chimeric antigen receptor T cell (CAR-T) immunotherapy has increasingly emerged as a promising therapeutic strategy for hematologic malignancies. Recent studies demonstrated that tucidinostat promoted the expression of CD22 on the B-cell tumor cells and enhanced the function of CD22 CAR-T (Yang X. et al., 2021). It was reported that low expression of phorbol-12-myristate-13-acetate-induced protein 1 (NOXA), a BH3-only BCL2 family protein, is an important biomarker for the drug resistance of CAR-T cell therapy, and NOXA can be transcriptionally activated by HDAC inhibitors (Yan et al., 2022). Therefore, the phase I/II study to evaluate whether tucidinostat improve clinical response to CAR-T in patients with R/R B-cell NHL is recruiting patients (NCT05370547).

#### 3.4.2 Solid tumors

Nivolumab, a PD1 inhibitor, has shown significant therapeutic effects in a wide range of cancers, including melanoma (MEL), lung cancer, and renal cell carcinoma (RCC). A phase Ib/2 study was conducted to evaluate the safety and efficacy of tucidinostat-nivolumab regimen in MEL, NSCLC, and RCC. The results demonstrated that tucidinostat in combination with nivolumab was well tolerated. The ORR was 67% (12, 18), 33% (3/9), 38% (3/8) for MEL, RCC and NSCLC respectively. Then the phase II trial in MEL was performed to show that the combination of tucidinostat and nivolumab was well tolerated and a 71% (24, 34) ORR was achieved. The encouraging efficacy, especially for MEL warrants for further clinical investigation of tucidinostat and nivolumab (NCT03074318) (Wagner, 2020).

Similar to targeted therapeutics, unresponsiveness and acquired resistance also occur during treatment with immune checkpoint inhibitors (ICIs) targeting PD1/PD-L1. Recent studies suggested that tucidinostat improved the efficacy of nivolumab through epigenetic modulations in the tumor environment, such as upregulation of major histocompatibility complex (MHC) I and II. The pre-clinical study provided strong rationale for the combination therapies in the clinic (Bissonnette et al., 2021).

Soft tissue sarcoma (STS) is a malignant tumor with poor prognosis and more than 40% of patients would develop metastasis diseases (Gamboa et al., 2020). Since effective chemotherapeutics and targeted therapy are limited for the treatment of STS, there is an urgent need to develop novel therapeutic approaches. It was reported that treatment with anti-PD-1 antibody pembrolizumab achieved an ORR less than 20% in patients with advanced sarcoma. Que et al. identified that the HDAC gene family was amplified in more than 70% of patients with lipocarcinoma. PD-1 expression was increased after tucidinostat addition through the activation of the transcriptional factor signal transducer and activator of transcription 1 (STAT1), accompanied by the infiltration of CD8^+^ T cells. Therefore, a rational combination strategy of tucidinostat and toripalimab was performed in a murine mouse model, with tumor regression and extended survival (Que et al., 2021). The combination of tucidinostat with anti-PD-L1 antibody was suggested to improve efficacy for the treatment of NSCLC and TNBC as well (Zhang W. et al., 2019; Tu et al., 2021). A multicenter, randomized, double-blind phase III study of tucidinostat in combination with nivolumab as a first-line therapy for melanoma in a phase III clinical trial in the US.

## 4 Safety and priority of tucidinostat

The safety and efficacy of tucidinostat as a single agent was first assessed in patients with advanced solid tumors and lymphomas in 2012. It was shown that no dose limiting toxicity (DLT) occurs in the group with 50 mg tucidinostat twice weekly (BIW) treatment for four consecutive weeks in a 6-week cycle, which indicated that tucidinostat is a safe and well-tolerated regimen (Dong et al., 2012).

The toxicity profile of tucidinostat was manageable, including thrombocytopenia, neutropenia, fatigue, nausea/vomiting, and anemia. Most adverse effects were of grade 1 to 2, which indicates tucidinostat as an effective therapy with an acceptable safety profile (Shi et al., 2017). The incidence of each adverse effect induced by tucidinostat was less than 30%, in contrast to another HDAC inhibitor, vorinostat treatment, where the incidence of each adverse effect is higher (diarrhea (48.6%), fatigue (45.9%) and nausea (25.7%)) (Lu et al., 2016; Shi et al., 2017).

Tucidinostat has shown priorities to other HDAC inhibitors, including lower side effects, higher clinical efficacy and convenience. Hydroxamates are pan-HDAC inhibitors with high incidence of side effect, while benzamide derivatives are isotype-selective HDAC inhibitors with lower toxicity profiles (Pan et al., 2014). Entinostat, the other oral benzamide-class HDAC inhibitor, showed significant biological activity in patients, but its efficacy as a single agent therapy remains limited (Connolly et al., 2017).

In comparison to HDAC pan-inhibitors, the subtype-selective HDAC inhibitor tucidinostat decrease and functionally inhibit the expansion of Treg and MDSC to overcome immune-suppressive tumor microenvironment (Que et al., 2021; Chen et al., 2022). These findings provide a rationale for combination regimens of tucidinostat and anti-PD-1 immunotherapy. As far as synergistic effect with anti-PD-1 is concerned, tucidinostat is superior and has a much wider clinical perspective than HDAC pan-inhibitors.

In terms of clinical treatment, the oral route of intake of tucidinostat provides higher convenience compared to other HDAC inhibitors, such as romidepsin, which is administered by injection. The price of tucidinostat is also much lower than that of other approved HDAC inhibitors, and cheaper than chemotherapy. Therefore, expanding the use of tucidinostat clinically could greatly improve the effectiveness of cancer treatment. Although tucidinostat is not approved for worldwide application, the clinical trial is currently ongoing in the United States (NCT02733380). In summary, tucidinostat, as a first-in-class benzamide HDAC inhibitor, exhibits great potential and priority for cancer therapy.

## 5 Conclusion and future perspectives

Tucidinostat, a novel and orally active benzamide class of HDAC inhibitor, exhibited high potency for the treatment of cancer and non-cancer diseases, such as the common autoimmune bleeding disorder ITP and AIDS as an antiretroviral therapy(Schmiegelow, 2019; Zhao et al., 2019; Li et al., 2020). In the cancer field, tucidinostat is currently being evaluated in many clinical trials with the most advanced research progress being in the field of hematological malignancies. In December 2014, tucidinostat was approved by the Chinese FDA as a second-line therapy for R/R PTCL as monotherapy or in combination regimen with the existing therapies. In June 2021, the Japanese MHLW approved tucidinostat for R/R PTCL and R/R AITL.

Tucidinostat as a monotherapeutic agent for DLBCL is being evaluated in a phase II clinical trial in Japan and in combination with nivolumab as a first-line therapy for melanoma in a phase III clinical trial in the US. The clinical effect of tucidinostat has been shown in R/R DLBCL as a monotherapy, especially in those with CREBBP mutation (Sun et al., 2021). Tucidinostat exhibits an ORR of 39.06% (Shi et al., 2017) and the efficacy as a monotherapy in PTCL is evident. PTCL patients of certain subtypes, especially AITL, shows prominent response rate to tucidinostat, which may be due to the important role of epigenetic regulation in AITL pathogenesis and the unique epigenetic modulating mechanisms of tucidinostat (Lu et al., 2016; Shi et al., 2017). It has also been used in MM, MDS, and B-LBL with varying degrees of efficacy, demonstrating the effectiveness of tucidinostat for hematological malignancies.

Other than monotherapy, tucidinostat as a combination regimen seems to be promising in the treatment of solid tumors, such as in combination with exemestane in advanced breast cancer. The most impressive efficacy is observed when tucidinostat was used in combination with immunotherapy, which significantly increased the efficacy of checkpoint inhibitors (CPIs) in both hematological and solid tumors. A number of ongoing clinical investigations examine tucidinostat use in various combinations for relapsed as well as newly diagnosed PTCL (Tables 3–5), showing that tucidinostat can produce synergistic effects with other drugs.

**TABLE 3:** Ongoing clinical trials of tucidinostat in combination with chemotherapy.

Num	Status	Study Title	Conditions	Drugs + tucidinostat	Phase	Estimated enrollment	NCT number	Study Start Date
1	Unknown	Tucidinostat With PET Regimen for Angioimmunoblastic T Cell Lymphoma (PET: Prednisone, Etoposide, and Thalidomide)	AITL	PET Regimen	Phase II	30	NCT03273452	1-Mar-17
2	Recruiting	Study Evaluating the Safety and Efficacy of C-CHOP in Untreated Subjects with Angioimmunoblastic T Cell Lymphoma	AITL	CHOP Regimen	Phase II	23	NCT03853044	29-Dec-18
3	Recruiting	Efficacy and Safety of Tucidinostat in CBF Leukemia	AML	Cytarabine	Phase I	250	NCT03031262	8-Feb-17
4	Unknown	Tucidinostat Plus DCAG for R/R AML	AML	DCAG regimen	Phase I	100	NCT02886559	Jun-16
5	Unknown	DCHA as Postremission Therapy for AML With t (8;21)	AML with t (8;21)	DCHA Regimen	Phase I	120	NCT03453255	1-Jan-18
6	Unknown	Tucidinostat Plus DICE Regimen for Patients with Relapse or Refractory B-cell Non-Hodgkin’s Lymphoma (NHL)	B-cell NHL	DICE Regimen	Phase II	46	NCT03105596	11-Apr-17
7	Unknown	Tucidinostat with R-CHOP Regimen for DLBCL Patients	DLBCL	R-CHOP regimen	Phase II	39	NCT03201471	20-Aug-17
8	Unknown	Tucidinostat Combined with Clad/Gem/Bu With AutoSCT in R/R Diffuse Large B Cell Lymphoma	DLBCL	Cladribine, gemcitabine	Phase II	93	NCT03151876	12-Jun-17
9	Recruiting	Phase III Study of Tucidinostat in Combination with R-CHOP in Patients with Newly Diagnosed Double-Expressor DLBCL	DLBCL	R-CHOP Regimen	Phase III	418	NCT04231448	21-May-20
10	Recruiting	Biomarker Guided Treatment in DLBCL	DLBCL	R-CHOP Regimen	Phase II	128	NCT04025593	17-Jul-19
11	Unknown	Tucidinostat Combined With R-GDP in Treating Patients With R/R Diffuse Large B-cell Lymphoma (DLBCL)	DLBCL	R-GDP Regimen	Phase II	63	NCT03373019	21-Dec-17
12	Recruiting	Tucidinostat + R-GemOx Regimen as Salvage Treatment for Transplant-ineligible Patients With R/R DLBCL	DLBCL	Rituximab, Gemcitabine,Oxaliplatin	Phase II	54	NCT04022005	15-Jul-19
13	Unknown	Tucidinostat Combined With VDDT Regimen in the Relapse and Refractory Diffuse Large B Cell Lymphoma	DLBCL	VDDT Regimen	Phase II	20	NCT02733380	May-16
14	Unknown	Efficacy and Safety of Chi-BEAC Combining With Auto-HSCT to Treat Aggressive Lymphoma Subjects	DLBCL, PTCL	Tucidinostat	Phase II	69	NCT03629873	1-Feb-18
15	Unknown	Tucidinostat Plus R-CHOP in Elderly DLBCL	Elderly DLBCL	R-CHOP	Phase II	49	NCT02753647	Apr-16
16	Unknown	Tucidinostat Combined With Clad/Gem/Bu With AutoSCT in High Risk Hodgkin and Non-Hodgkin Lymphoma	HL, NHL	Cladribine, gemcitabine	Phase II	30	NCT03602131	1-Jan-19
17	Recruiting	Comparation of Tucidinostat Plus VRD (Bortezomib, Lenalidomide, Dexamethasone) With VRD Regimen for Primary High-Risk Multiple Myeloma Patients	MM	VRD Regimen	Phase I	50	NCT04025450	15-Jul-19
18	Not yet recruiting	Phase II Trial of Tucidinostat-Lenalidomide-Dexamethasone (CRD) Regimen in R/R MM	MM	Lenalidomide, Dexamethasone	Phase II	25	NCT03605056	31-Jul-18
19	Unknown	Tucidinostat With CHOP Regimen for *de novo* PTCL Patients (CHOP: Cyclophosphamide, Etoposide, Vincristine and Prednisone; PTCL: Peripheral T Cell Lymphoma)	PTCL	CHOP Regimen	Not Applicable	39	NCT03268889	15-Jun-17
20	Unknown	Tucidinostat With ICE Regimen for R/R Peripheral T Cell Lymphoma	PTCL	ICE regimen	Phase II	35	NCT02856997	Sep-16
21	Not yet recruiting	Clinical Study of Mitoxantrone Hydrochloride Liposome Injection vs. Tucidinostat in Patients With R/R PTCL	PTCL	Mitoxantrone Hydrochloride Liposome	Phase III	190	NCT04668690	1-Jan-21
22	Completed	Clinical Trial of Tucidinostat Combined With CHOP in Peripheral T-cell Lymphoma Patients	PTCL	CHOP Regimen	Phase I	30	NCT02809573	11-Aug-16
23	Unknown	Compared the Efficacy and Safety of CDOP Combined With Tucidinostat and CDOP in de Novo Peripheral T Cell Lymphoma Patients	PTCL	CDOP Regimen	Phase III	174	NCT03023358	Feb-17
24	Recruiting	Azacitidine Combined With Tucidinostat in the Treatment of Newly Diagnosed PTCL Unfit for Conventional Chemotherapy	PTCL	Azacitidine	Phase II	28	NCT04480125	20-Jun-20
25	Unknown	Tucidinostat Combined With PECM in R/R Peripheral T-cell Lymphoma (PTCL)	PTCL	PECM Regimen	Phase II	102	NCT03321890	7-Mar-17
26	Recruiting	Tucidinostat Combined With CHOPE Regimen for Peripheral T-cell Lymphoma Patients	PTCL	CHOPE Regimen	Phase II	114	NCT03617432	28-Aug-18
27	Recruiting	Targeted Drug Combined With CHOP in the Treatment of Newly Diagnosed Peripheral T-cell Lymphoma	PTCL	CHOP Regimen	Phase II	106	NCT04480099	20-Jun-20
28	Recruiting	Tucidinostat Plus CHOEP Combined With Upfront ASCT in Untreated Peripheral T-cell Lymphoma	PTCL	CHOPE Regimen	Phase I	100	NCT02987244	Mar-16
29	Unknown	Tucidinostat Combined With Cyclophosphamide, Prednisone, Thalidomide in Treatment of Fragile Patients With Relapse/Refratory Peripheral T Cell Lymphoma	PTCL	CPT Regimen	Phase II	45	NCT02879526	Aug-16
30	Unknown	Tucidinostat Combined With Cyclophosphamide, Prednisone, Thalidomide in Treatment of Fragile Patients With Relapse/Refratory Peripheral T Cell Lymphoma	PTCL	C-CPT	Phase II	45	NCT02879526	Aug-16
31	Active, not recruiting	CDIAG Regimen in the Treatment of R/R Acute Myeloid Leukemia	R/R AML	CDIAG regimen	Phase II	40	NCT03985007	1-Jan-18
32	Recruiting	New Double Epigenetic Regimen in the Treatment of R/R Acute Myeloid Leukemia	R/R AML	CAHAG regimen	Phase II	219	NCT05029141	1-Sep-21
33	Completed	Tucidinostat in Combination With Carboplatin and Paclitaxel in Advanced Non-small Cell Lung Cancer	NSCLC	Carboplatin + Paclitaxel	Phase II	124	NCT01836679	Apr-13
34	Recruiting	Tucidinostat Plus Etoposide and Cisplatin/Carboplatin as First-line Treatment for Extrapulmonary Neuroendocrine Carcinoma	Neuroendocrine Tumors	Cisplatin/carboplatin	Phase II	28	NCT05076786	27-Oct-21
35	Completed	Tucidinostat Combined With Cisplatin in Head and Neck Adenoid Cystic Carcinoma (HNACC)	Adenoid Cystic Carcinomas	Cisplatin	Phase II	22	NCT03639168	6-Jun-18
36	Recruiting	Neoadjuvant Treatment of Early Triple-negative Breast Cancer With Tucidinostat and Chemotherapy	Triple-negative Breast Cancer	Docetaxel + Epirubicin	Not Applicable	20	NCT04582955	30-Oct-20
37	Not yet recruiting	Tucidinostat Combined With Cisplatin for Relapsed or Metastatic Triple-negative Breast Cancer	TNBC	Cisplatin	Phase II	55	NCT04192903	25-Dec-19
38	Recruiting	Study of Tucidinostat Combined With Cladribine in R/R Acute Myeloid Leukemia	AML	Cladribine	Phase II	31	NCT05330364	1-Jun-21
39	Not yet recruiting	Efficacy and Safety of Tucidinostat Combined With BEAM Pretreatment Regimen in Autologous Transplantation for T-cell Lymphoma: a Single-center, Single-arm Clinical Study	T-cell Lymphoma	BEAM	Phase II	23	NCT05367856	1-Jun-22
40	Recruiting	Clinical Study of Tucidinostat Combined With Chemotherapy in Neoadjuvant Treatment of HR+/HER2-BC	Breast Cancer	pharmorubicin, Cyclophosphamide, Docetaxel	Phase II	59	NCT05400993	2 June 2022
41	Not yet recruiting	Tucidinostat Combined With Exemestane (± Goserelin) Versus Neoadjuvant Chemotherapy in Patients of Stage II-III HR-positive/HER2-negative Breast Cancer	Breast Neoplasms	docetaxel, epirubicin	Phase II, III	130	NCT05253066	23-Feb-22
42	Not yet recruiting	Tucidinostat and Metronomic Capecitabine for Metastatic Triple-negative Breast Cancer:a Multicenter, Open-label, Randomized Controlled, Phase II Clinical Trial	TNBC	Capecitabine	Phase II	107	NCT05390476	1-Aug-22
43	Recruiting	Tucidinostat Plus Etoposide in the Treatment of Neuroblastoma in Childhood.	Neuroblastoma in Children	etoposide	Phase I	30	NCT05338541	27-May-22
44	Not yet recruiting	Eribulin Plus Tucidinostat in the Treatment of HER2-negative Advanced Breast Cancer	HER2 Negative Breast Cancer	Eribulin	Phase I, II	102	NCT05335473	1-Jul-22

CBF, Core binding factor; AutoSCT, Autologous stem cell transplantation; DCAG, Decitabine, cytarabine, aclarubicin hydrochloride and granulocyte colony-stimulating factor; DCHA, Decitabine, cladribine, homoharringtonine, and cytarabine; DICE, Dexamethasone, ifosfamide, cisplatin, etoposide; R-GDP, Rituximab, gemcitabine, dexamethasone, cisplatin; VDDT, Vinorelbine,liposomal doxorubicin,dexamethasone and thalidomide; BEAC, Carmustine, etoposide, cytarabine, and cyclophosphamide; ICE, Ifosfamide, carboplatin and etoposide; CDOP, Cyclophosphamide, doxorubicin, vincristine, prednisone; PECM, Prednisone, etoposide, cyclophosphamide and methotrexate; CHOPE, Cyclophosphamide, doxorubicin, vincristine, prednisone and etoposide; CDIAG, Tucidinostat, decitabine, cytosine arabinoside, and granulocyte-colony stimulating factor (G-CSF); CAHAG, Tucidinostat, azacytidine, homoharringtonine, cytarabine, G-CSF; BEAM, Carmustine, Etoposide, Cytarabine and Melphalan; TNBC, Triple-negative breast cancer.

**TABLE 4:** Ongoing clinical trials of tucidinostat in combination with targeted therapy.

Num	Status	Study Title	Conditions	Drugs + tucidinostat	Phase	Estimated enrollment	NCT number	Study start date
1	Unknown	Precision Diagnosis Directing HDACi and TKI Target Therapy for Adult Ph-like ALL	Adult Ph-like ALL	TKI	Phase II	120	NCT03564470	14-Feb-16
2	Recruiting	Rituximab Combined With Tucidinostat and Lenalidomide for r/r AITL	AITL	Rituximab	Not Applicable	26	NCT04319601	13-Mar-20
3	Recruiting	Ruxolitinib and Tucidinostat for Acute T Cell Lymphoblast Leukemia/Lymphoblastic Lymphoma	ALL	Ruxolitinib	Phase I	50	NCT05075681	1-Nov-21
4	Recruiting	Ruxolitinib and Tucidinostat Intensified Bu/CY Conditioning Regimen	ALL	Ruxolitinib	Phase II	50	NCT05088226	1-Dec-21
5	Not yet recruiting	A Phase II Trail of Tucidinostat, Rituximab and Methotrexate in Lymphoma Patients	Central Nervous System Lymphoma	Rituximab, methotrexate	Phase II	51	NCT04516655	1-Sep-20
6	Not yet recruiting	Tofacitinib Combined With Tucidinostat in R/R ENKTCL	ENKTCL	Tofacitinib	Phase II	20	NCT03598959	1-Jan-19
7	Recruiting	Addition of Tucidinostat to the Combination Treatment of Decitabine Plus Camrelizumab in Combination Treatment Resistant/Relapsed Patients With Classical Hodgkin Lymphoma	HL	Decitabine, Camrelizumab	Phase II	100	NCT04233294	2-Feb-20
8	Recruiting	Chiauranib in Combination With Tucidinostat in Patients With R/R Non-Hodgkin’s Lymphoma	NHL	Chiauranib	Phase I	24	NCT03974243	31-Dec-21
9	Recruiting	Tucidinostat in Combination With Decitabine in Non-Hodgkin’s Lymphoma Relapsed After Chimeric Antigen Receptor	NHL	Decitabine, Camrelizumab	Phase I	100	NCT04337606	4-Apr-20
10	Recruiting	A Clinical Trial of Tucidinostat Combined With Etoposide in R/R NK/T-cell Lymphoma	NKTL	Etoposide	Phase IV	30	NCT04490590	1-Oct-16
11	Not yet recruiting	PI3Kδ Inhibitor Parsaclisib Combined With Tucidinostat for the Treatment of R/R Peripheral T-cell Lymphoma	PTCL	Parsaclisib	Phase I	28	NCT05083208	Jan-22
12	Recruiting	Tucidinostat Combination With Lenalidomide in Patients With R/R Peripheral T-cell Lymphoma	PTCL	Lenalidomide	Phase II	44	NCT04329130	27-Mar-20
13	Active, not recruiting	Trial of Tucidinostat in Combination With Exemestane in Patients With Advanced Breast Cancer	Breast Cancer	exemestane	Phase III	365	NCT02482753	Jul-15
14	Recruiting	Clinical Study of Tucidinostat Combined With Fulvestrant in the Treatment of Hormone Receptor-positive Advanced Breast Cancer	Breast Cancer	Fulvestrant	Not Applicable	82	NCT05047848	18-Aug-21
15	Recruiting	Neoadjuvant Tucidinostat and Exemestane in Early Breast Cancer	Breast Cancer	Tucidinostat, exemestane	Phase II	30	NCT04465097	8-Jul-20
16	Not yet recruiting	Tucidinostat and Fulvestrant in Hormone-receptor Positive Advanced Breast Cancer	Breast Cancer	Fulvestrant	Phase II	73	NCT04999540	1-Nov-21
17	Unknown †	Tucidinostat With EGFR-TKI for Advanced EGFR-TKI-resistant Non-Small Cell Lung Cancer	NSCLC	EGFR-TKI	Phase II	20	NCT02815007	Jun-16
18	Recruiting	Phase II Umbrella Study Directed by Next Generation Sequencing	NSCLC	Afatinib	Phase II	400	NCT03574402	9-Jul-18
19	Not yet recruiting	Azacytidine Plus Tucidinostat in the Treatment of Relapsed and Refractory Angioimmunoblastic T-cell Lymphoma	AITL	Azacitidine	Phase II	20	NCT05179213	1-Jan-22
20	Recruiting	Single Arm Study of Post-transplant Azacitidine and Tucidinostat for Prevention of Acute Myelogenous Leukemia Relapse	AML	Azacitidine	Phase1, 2	20	NCT05270200	1-Feb-22
21	Recruiting	Tucidinostat in Combination With Abemaciclib and Fulvestrant in Breast Cancer Patients Previously Treated With Palbociclib	Breast Cancer	Abemaciclib, Fulvestrant	Phase I, 2	44	NCT05464173	1-Jul-22
22	Not yet recruiting	Venetoclax Combining Tucidinostat and Azacitidine (VCA) in the Treatment of R/R AML	R/R AML	venetoclax, azacitidine	Phase II	30	NCT05305859	1-Apr-22
23	Not yet recruiting	An Exploratory Study of Surufatinib Combined With Tucidinostat and Fulvestrant in HR Positive Unresectable Metastatic Breast Cancer	Breast cancer	surufatinib, fulvestrant	Phase II	63	NCT05186545	Jul-22
24	Not yet recruiting	Clinical Study of Fulvestrant Combined With Tucidinostat in the Treatment of Hormone Receptor-positive Advanced Breast Cancer Resistant to CDK4/6 Inhibitors	Breast Cancer	Fulvestrant	Phase IV	20	NCT05191914	7-Feb-22
25	Not yet recruiting	Tucidinostat, Azacitidine Combined With CHOP Versus CHOP in Patients With Untreated Peripheral T-cell Lymphoma	PTCL	Azacitidine, CHOP	Phase III	87	NCT05075460	1-Oct-21

TKI, Tyrosine kinase inhibitors; ENKTCL, Extranodal natural killer/T-Cell lymphoma; NSCLC, Non-small-cell lung carcinoma; AITL, Angioimmunoblastic T-cell lymphoma: AML, Acute myeloid leukemia.

**TABLE 5:** Ongoing clinical trials of tucidinostat in combination with immunotherapy.

Num	Status	Study Title	Conditions	Drugs + tucidinostat	Phase	Estimated enrollment	NCT number	Study start date
1	Recruiting	Anti-PD-1 Antibody Combined With Peg-Asparaginase and Tucidinostat for the Early Stage of NK/T Cell Lymphoma	NKTL, Early Stage	Anti-PD-1 antibody + Peg-Asparaginase	Phase II	35	NCT04414969	26/6/2020
2	Not yet recruiting	Sintilimab in Combination With Tucidinostat in R/R AITL	AITL	Sintilimab	Phase II	83	NCT04831710	15/4/2021
3	Recruiting	Sintilimab in Combination With Tucidinostat in R/R ENKTCL	R/R ENKTCL	Sintilimab	Phase I	50	NCT03820596	29/3/2019
4	Not yet recruiting	Sintilimab in Combination With Tucidinostat in Newly Diagnosed ENKTCL	Newly Diagnosed ENKTCL	Sintilimab	Phase II	30	NCT04994210	1/9/2021
5	Not yet recruiting	Anti-PD-1 Antibody Plus Tucidinostat and Rituximab Regimen in R/R DLBCL (PCR)	R/R DLBCL	Anti-PD-1 Antibody + Rituximab	Phase II	27	NCT05115409	1/12/2021
6	Recruiting	PD-1 Antibody, Tucidinostat, Lenalidomide and Etoposide for R/R NK/T Cell Lymphoma	NKTL	PD-1 Antibody + lenalidomide + etoposide	Phase IV	50	NCT04038411	1/4/2019
7	Recruiting	Sintilimab Combined With Tucidinostat in Treating Peripheral T Cell Lymphoma	PTCL	Sintilimab	Phase II	51	NCT04512534	13/11/2020
8	Recruiting	Sintilimab Plus Tucidinostat in the Treatment of Relapsed and Refractory Cutaneous T-cell Lymphoma: a Multicenter Phase II Study	CTCL	Sintilimab	Phase II	52	NCT04296786	1/11/2019
9	Recruiting	PD-1 Antibody, Tucidinostat, Lenalidomide and Gemcitabine for Peripheral T-cell Lymphoma	PTCL	PD-1 antibody + lenalidomide + gemcitabine	Phase IV	100	NCT04040491	1/9/2019
10	Recruiting	The Clinical Trial of Tucidinostat + Decitabine + Camrelizumab Versus Decitabine + Camrelizumab in Anti-PD-1 Antibody Resistant Patients With Classical Hodgkin Lymphoma.	HL	Decitabine + Camrelizumab	Phase II	200	NCT04514081	1/8/2020
11	Not yet recruiting	Sintilimab (IBI308) in Combination With Tucidinostat and Azacitidine in Refractory or Relapsed PTCL	PTCL	Sintilimab	Phase II	30	NCT04052659	15/4/2021
12	Not yet recruiting	Induction Chemotherapy Sequential Sintilimab Combined With Dual Epigenetic Drugs for ENKTL-HLH	ENKTL-HLH	Sintilimab + Azacitidine	Phase II	37	NCT05008666	1/12/2021
13	Recruiting	Decitabine-primed Tandem CD19/CD20 CAR T Cells Plus Epigenetic Agents in Aggressive r/r B-NHL With Huge Tumor Burden	R/R B-NHL	Decitabine-primed Tandem CAR19/20 engineered T cells	Phase I,II	80	NCT04553393	9/9/2020
14	Recruiting	Tucidinostat With Immunotherapy for Patients With Locally Advanced or Metastatic Urothelial Carcinoma	Bladder Cancer Stage IV	tislelizumab	Phase II	43	NCT04562311	1/10/2020
15	Recruiting	A Single-arm, Open, Phase II Study of Tucidinostat Combined With Toripalimab in Refractory and Advanced Soft-tissue Sarcoma	Soft-tissue Sarcoma	Toripalimab	Phase II	53	NCT04025931	19/1/2020
16	Not yet recruiting	Tucidinostat Plus Camrelizumab as Second-line Therapy for Advanced ESCC Treated With PD-1 Blockade	ESCC	camrelizumab	Phase II	73	NCT04984018	1/12/2021
17	Recruiting	Tucidinostat Plus Sintilimab for Chemotherapy-refractory Advanced High-grade Neuroendocrine Neoplasm	Neuroendocrine Tumors	Sintilimab	Phase II	23	NCT05113355	17/11/2021
18	Not yet recruiting	Trial of Tucidinostat in Combination With Envafolimab in Patients With PD-1 Inhibitor Resistant Advanced NSCLC.	NSCLC	Envafolimab	Phase II	69	NCT05068427	1/11/2021
19	Recruiting	Anti-PD-1 Antibody Combined With Histone Deacetylase Inhibitor in Patients With Advanced Cervical Cancer	Cervical Cancer	Toripalimab	Phase I	40	NCT04651127	9/11/2020
20	Not yet recruiting	A Study of Sintilimab and Tucidinostat in Combination With or Without IBI305 in Standard Treatment Failure of Advanced or Metastatic pMMR/MSS Colorectal Carcinoma	Advanced Microsatellite Stable Colorectal Cancer	Sintilimab	Phase II	48	NCT04724239	1/2/2021
21	Active, not recruiting	Study of HBI-8000 With Nivolumab in Melanoma, Renal Cell Carcinoma and Non-Small Cell Lung Cancer	Melanoma, Renal Cell Carcinoma, NSCLC	Nivolumab	Phase I, II	96	NCT02718066	1/8/2016
22	Not yet recruiting	A Study of HBI-8000 (Tucidinostat) With Pembrolizumab in Non-Small Cell Lung Cancer	NSCLC	Pembrolizumab	Phase II	24	NCT05141357	1/12/2021
23	Recruiting	Tucidinostat Bridging for CAR-T Therapy	NHL	tucidinostat, Anti-CD19 CAR-T cells	Phase I, II	120	NCT05370547	25/5/2022
24	Recruiting	Study of Tucidinostat, Decitabine and Immune Checkpoint Inhibitors in R/R NHL and Advanced Solid Tumors	R/R NHL, advanced solid tumors	Decitabine and Immune Checkpoint Inhibitors	Phase I, II	100	NCT05320640	11-Apr-22
25	Not yet recruiting	A Clinical Study of the Efficacy and Safety of Tucidinostat in Combination With Camrelizumab and Carboplatin or Capecitabine in the Second and Third Line Treatment of Relapsed/Metastatic Triple-negative Breast Cancer	TNBC	carboplatin, capecitabine	Phase I	70	NCT05438706	10-Jul-22
26	Not yet recruiting	Clinical Study of Tucidinostat Combined With Toripalimab in the Treatment of Advanced Melanoma	Melanoma	Toripalimab	Phase I	43	NCT05478473	Aug-22
27	Recruiting	Tucidinostat and PD-1 Inhibitor for Advanced Esophagus Cancer, AEG, Gastric Cancer	Esophageal Squamous Cell Cancer, AEG, Gastric Adenocarcinoma	Toripalimab	Phase II	87	NCT05163483	1-Jul-22

PTCL, peripheral T-cell lymphoma; ENKTL-HLH, extranodal NK/T cell lymphoma with hemophagocytic lymphohistiocytosis; ESCC, Esophageal squamous cell carcinoma; pMMR /MSS, Proficient mismatch repair /microsatellite stable; AEG, Esophagastric Junction Cancers; CAR-T, Chimeric antigen receptor T cell.

In summary, tucidinostat shows favorable therapeutic efficacy alone or in combination to treat a wide variety of cancers. In the future, new combined regimens could be further investigated, especially the synergistic effect of tucidinostat with immunotherapy could be a potential option for multiple malignancies, including for lymphoma, melanoma, and breast cancer. Currently, immunotherapy is prominent treatment option with high response rate for different types of cancers, while in combination with tucidinostat was shown to remarkably improve the ORR by 10–20% (Nie et al., 2019; Wang C. et al., 2021). More clinical trials could be conducted to explore the effectiveness of the combination between tucidinostat and different immunotherapies and to understand the underlying molecular mechanisms of epigenetic regulation of immunogenicity by tucidinostat. In addition, further studies to identify potential biomarkers for specific therapies through high throughput omics are warranted. The elucidation of predictive biomarkers and molecular mechanisms for the combination regimen of CPIs and tucidinostat would provide rationale and promote precision medication for clinical application.

## Glossary

ACC: Adenoid cystic carcinoma
AIDS: Acquired immune deficiency syndrome
AML: Acute myeloid leukemia
ALK: Anaplastic lymphoma kinase
ASCT: Autologous stem cell transplant
ATLL: Adult T-cell leukemia-lymphoma
BIW: Twice per week
BTG1: B-cell translocation gene 1
BTZ: Bortezomib
CFDA: China Food and Drug Administration
cGVHD: Chronic graft-versus-host disease
CSCO: Chinese Society of Clinical Oncology
ctDNA: Circulating tumor DNA
CR: Complete response
DCR: Disease control rate
DEL: Double-expresser lymphoma
DLT: Dose limiting toxicity
DNMT: DNA methyltransferase
EGFR-TKI: Epidermal growth factor receptor tyrosine kinase inhibitor
ENKTL: Extranodal natural killer/T cell lymphoma
GOF: Gain-of function
HAT: Histone acetyltransferases
HDACHistone deacetylase
HDACis: Histone deacetylases inhibitors
ICIs: Immune checkpoint inhibitors
IMRT: Intensity-modulated radiation therapy
ITP: Idiopathic thrombocytopenic purpura
LOF: Loss-of-function
MDR: Multi-drug resistance
MDS: Myelodysplastic syndromes
MEL: Melanoma
MHLW: Ministry of Health, Labour and Welfare
MM: Multiple myeloma
NHL: Non-Hodgkin’s lymphoma
NKT: Natural killer/T-cell lymphoma
NMPA: National Medical Products Administration
NSCLC: Non-small cell lung cancer
ORR: Overall response rate
PD-L1: Programmed cell death ligand 1
PR: Partial response
PTCL: Peripheral T-cell lymphoma
PTCL-NOS: Peripheral T-cell lymphomas, not otherwise specified
QD: Every day
RCC: Renal cell carcinoma
ROS: reactive oxidative stress
SDHA: Succinate dehydrogenase subunit A
SIRT1: Sirtuin 1
STS: Soft tissue sarcoma
TIW: Three times per week
TNBC: Triple-negative breast cancer
T-LBL: T-cell lymphoblastic lymphoma
WES: Whole exome sequencing
